# Supplementary material for: HIVprotI: an integrated web based platform for prediction and design of HIV proteins inhibitors
Source: J Cheminform. 2018 Mar 9;10:12. doi: 10.1186/s13321-018-0266-y (PMC5845081; doi:10.1186/s13321-018-0266-y)
Supplement: Supplementary file 2 — Additional file 2. Source code of HIVProtI web server. [file 13321_2018_266_MOESM2_ESM.zip › HIVprotI_Source-code/hivprotI/weka-3-6-6/doc/weka/classifiers/trees/j48/Distribution.html]

Distribution


---


|  |  |  |
| --- | --- | --- |
| **Overview** \| **Package** \| **Class** \| **Tree** \| **Deprecated** \| **Index** \| **Help** \| **Weka's home** | |  |
| **PREV CLASS**   **NEXT CLASS** | **FRAMES**    **NO FRAMES**     **All Classes** |  |
| SUMMARY: NESTED \| FIELD \| CONSTR \| METHOD | DETAIL: FIELD \| CONSTR \| METHOD |  |


---


## weka.classifiers.trees.j48 Class Distribution

```
java.lang.Object
  weka.classifiers.trees.j48.Distribution
```

**All Implemented Interfaces:**: java.io.Serializable, java.lang.Cloneable, RevisionHandler

---

``` public class Distribution extends java.lang.Object implements java.lang.Cloneable, java.io.Serializable, RevisionHandler ```

Class for handling a distribution of class values.

**Version:**
:   $Revision: 1.12 $

**Author:**
:   Eibe Frank (eibe@cs.waikato.ac.nz)

**See Also:**: Serialized Form

---

**Constructor Summary**

`Distribution(Distribution toMerge)`
  
          Creates distribution with only one bag by merging all
bags of given distribution.

`Distribution(Distribution toMerge,
int index)`
  
          Creates distribution with two bags by merging all bags apart of
the indicated one.

`Distribution(double[][] table)`
  
          Creates and initializes a new distribution using the given
array.

`Distribution(Instances source)`
  
          Creates a distribution with only one bag according
to instances in source.

`Distribution(Instances source,
ClassifierSplitModel modelToUse)`
  
          Creates a distribution according to given instances and
split model.

`Distribution(int numBags,
int numClasses)`
  
          Creates and initializes a new distribution.


| **Method Summary** | |
| --- | --- |
| `int` | `actualNumBags()`             Returns number of non-empty bags of distribution. |
| `int` | `actualNumClasses()`             Returns number of classes actually occuring in distribution. |
| `int` | `actualNumClasses(int bagIndex)`             Returns number of classes actually occuring in given bag. |
| `void` | `add(int bagIndex, double[] counts)`             Adds counts to given bag. |
| `void` | `add(int bagIndex, Instance instance)`             Adds given instance to given bag. |
| `void` | `addInstWithUnknown(Instances source, int attIndex)`             Adds all instances with unknown values for given attribute, weighted according to frequency of instances in each bag. |
| `void` | `addRange(int bagIndex, Instances source, int startIndex, int lastPlusOne)`             Adds all instances in given range to given bag. |
| `void` | `addWeights(Instance instance, double[] weights)`             Adds given instance to all bags weighting it according to given weights. |
| `boolean` | `check(double minNoObj)`             Checks if at least two bags contain a minimum number of instances. |
| `java.lang.Object` | `clone()`             Clones distribution (Deep copy of distribution). |
| `void` | `del(int bagIndex, Instance instance)`             Deletes given instance from given bag. |
| `void` | `delRange(int bagIndex, Instances source, int startIndex, int lastPlusOne)`             Deletes all instances in given range from given bag. |
| `java.lang.String` | `dumpDistribution()`             Prints distribution. |
| `java.lang.String` | `getRevision()`             Returns the revision string. |
| `void` | `initialize()`             Sets all counts to zero. |
| `double` | `laplaceProb(int classIndex)`             Returns relative frequency of class over all bags with Laplace correction. |
| `double` | `laplaceProb(int classIndex, int intIndex)`             Returns relative frequency of class for given bag. |
| `double[][]` | `matrix()`             Returns matrix with distribution of class values. |
| `int` | `maxBag()`             Returns index of bag containing maximum number of instances. |
| `int` | `maxClass()`             Returns class with highest frequency over all bags. |
| `int` | `maxClass(int index)`             Returns class with highest frequency for given bag. |
| `int` | `numBags()`             Returns number of bags. |
| `int` | `numClasses()`             Returns number of classes. |
| `double` | `numCorrect()`             Returns perClass(maxClass()). |
| `double` | `numCorrect(int index)`             Returns perClassPerBag(index,maxClass(index)). |
| `double` | `numIncorrect()`             Returns total-numCorrect(). |
| `double` | `numIncorrect(int index)`             Returns perBag(index)-numCorrect(index). |
| `double` | `perBag(int bagIndex)`             Returns number of (possibly fractional) instances in given bag. |
| `double` | `perClass(int classIndex)`             Returns number of (possibly fractional) instances of given class. |
| `double` | `perClassPerBag(int bagIndex, int classIndex)`             Returns number of (possibly fractional) instances of given class in given bag. |
| `double` | `prob(int classIndex)`             Returns relative frequency of class over all bags. |
| `double` | `prob(int classIndex, int intIndex)`             Returns relative frequency of class for given bag. |
| `void` | `shift(int from, int to, Instance instance)`             Shifts given instance from one bag to another one. |
| `void` | `shiftRange(int from, int to, Instances source, int startIndex, int lastPlusOne)`             Shifts all instances in given range from one bag to another one. |
| `void` | `sub(int bagIndex, Instance instance)`             Subtracts given instance from given bag. |
| `Distribution` | `subtract(Distribution toSubstract)`             Subtracts the given distribution from this one. |
| `double` | `total()`             Returns total number of (possibly fractional) instances. |

**Methods inherited from class java.lang.Object**

`equals, getClass, hashCode, notify, notifyAll, toString, wait, wait, wait`

**Constructor Detail**

### Distribution

```
public Distribution(int numBags,
                    int numClasses)
```

:   Creates and initializes a new distribution.

---


### Distribution

```
public Distribution(double[][] table)
```

:   Creates and initializes a new distribution using the given
    array. WARNING: it just copies a reference to this array.

---


### Distribution

```
public Distribution(Instances source)
             throws java.lang.Exception
```

:   Creates a distribution with only one bag according
    to instances in source.

    **Throws:**: `java.lang.Exception` - if something goes wrong

---


### Distribution

```
public Distribution(Instances source,
                    ClassifierSplitModel modelToUse)
             throws java.lang.Exception
```

:   Creates a distribution according to given instances and
    split model.

    **Throws:**: `java.lang.Exception` - if something goes wrong

---


### Distribution

```
public Distribution(Distribution toMerge)
```

:   Creates distribution with only one bag by merging all
    bags of given distribution.

---


### Distribution

```
public Distribution(Distribution toMerge,
                    int index)
```

:   Creates distribution with two bags by merging all bags apart of
    the indicated one.


**Method Detail**

### actualNumBags

```
public final int actualNumBags()
```

:   Returns number of non-empty bags of distribution.

---


### actualNumClasses

```
public final int actualNumClasses()
```

:   Returns number of classes actually occuring in distribution.

---


### actualNumClasses

```
public final int actualNumClasses(int bagIndex)
```

:   Returns number of classes actually occuring in given bag.

---


### add

```
public final void add(int bagIndex,
                      Instance instance)
               throws java.lang.Exception
```

:   Adds given instance to given bag.

    :   **Throws:**: `java.lang.Exception` - if something goes wrong

---


### sub

```
public final void sub(int bagIndex,
                      Instance instance)
               throws java.lang.Exception
```

:   Subtracts given instance from given bag.

    :   **Throws:**: `java.lang.Exception` - if something goes wrong

---


### add

```
public final void add(int bagIndex,
                      double[] counts)
```

:   Adds counts to given bag.

---


### addInstWithUnknown

```
public final void addInstWithUnknown(Instances source,
                                     int attIndex)
                              throws java.lang.Exception
```

:   Adds all instances with unknown values for given attribute, weighted
    according to frequency of instances in each bag.

    :   **Throws:**: `java.lang.Exception` - if something goes wrong

---


### addRange

```
public final void addRange(int bagIndex,
                           Instances source,
                           int startIndex,
                           int lastPlusOne)
                    throws java.lang.Exception
```

:   Adds all instances in given range to given bag.

    :   **Throws:**: `java.lang.Exception` - if something goes wrong

---


### addWeights

```
public final void addWeights(Instance instance,
                             double[] weights)
                      throws java.lang.Exception
```

:   Adds given instance to all bags weighting it according to given weights.

    :   **Throws:**: `java.lang.Exception` - if something goes wrong

---


### check

```
public final boolean check(double minNoObj)
```

:   Checks if at least two bags contain a minimum number of instances.

---


### clone

```
public final java.lang.Object clone()
```

:   Clones distribution (Deep copy of distribution).

    :   **Overrides:**: `clone` in class `java.lang.Object`

---


### del

```
public final void del(int bagIndex,
                      Instance instance)
               throws java.lang.Exception
```

:   Deletes given instance from given bag.

    :   **Throws:**: `java.lang.Exception` - if something goes wrong

---


### delRange

```
public final void delRange(int bagIndex,
                           Instances source,
                           int startIndex,
                           int lastPlusOne)
                    throws java.lang.Exception
```

:   Deletes all instances in given range from given bag.

    :   **Throws:**: `java.lang.Exception` - if something goes wrong

---


### dumpDistribution

```
public final java.lang.String dumpDistribution()
```

:   Prints distribution.

---


### initialize

```
public final void initialize()
```

:   Sets all counts to zero.

---


### matrix

```
public final double[][] matrix()
```

:   Returns matrix with distribution of class values.

---


### maxBag

```
public final int maxBag()
```

:   Returns index of bag containing maximum number of instances.

---


### maxClass

```
public final int maxClass()
```

:   Returns class with highest frequency over all bags.

---


### maxClass

```
public final int maxClass(int index)
```

:   Returns class with highest frequency for given bag.

---


### numBags

```
public final int numBags()
```

:   Returns number of bags.

---


### numClasses

```
public final int numClasses()
```

:   Returns number of classes.

---


### numCorrect

```
public final double numCorrect()
```

:   Returns perClass(maxClass()).

---


### numCorrect

```
public final double numCorrect(int index)
```

:   Returns perClassPerBag(index,maxClass(index)).

---


### numIncorrect

```
public final double numIncorrect()
```

:   Returns total-numCorrect().

---


### numIncorrect

```
public final double numIncorrect(int index)
```

:   Returns perBag(index)-numCorrect(index).

---


### perClassPerBag

```
public final double perClassPerBag(int bagIndex,
                                   int classIndex)
```

:   Returns number of (possibly fractional) instances of given class in
    given bag.

---


### perBag

```
public final double perBag(int bagIndex)
```

:   Returns number of (possibly fractional) instances in given bag.

---


### perClass

```
public final double perClass(int classIndex)
```

:   Returns number of (possibly fractional) instances of given class.

---


### laplaceProb

```
public final double laplaceProb(int classIndex)
```

:   Returns relative frequency of class over all bags with
    Laplace correction.

---


### laplaceProb

```
public final double laplaceProb(int classIndex,
                                int intIndex)
```

:   Returns relative frequency of class for given bag.

---


### prob

```
public final double prob(int classIndex)
```

:   Returns relative frequency of class over all bags.

---


### prob

```
public final double prob(int classIndex,
                         int intIndex)
```

:   Returns relative frequency of class for given bag.

---


### subtract

```
public final Distribution subtract(Distribution toSubstract)
```

:   Subtracts the given distribution from this one. The results
    has only one bag.

---


### total

```
public final double total()
```

:   Returns total number of (possibly fractional) instances.

---


### shift

```
public final void shift(int from,
                        int to,
                        Instance instance)
                 throws java.lang.Exception
```

:   Shifts given instance from one bag to another one.

    :   **Throws:**: `java.lang.Exception` - if something goes wrong

---


### shiftRange

```
public final void shiftRange(int from,
                             int to,
                             Instances source,
                             int startIndex,
                             int lastPlusOne)
                      throws java.lang.Exception
```

:   Shifts all instances in given range from one bag to another one.

    :   **Throws:**: `java.lang.Exception` - if something goes wrong

---


### getRevision

```
public java.lang.String getRevision()
```

:   Returns the revision string.

    :   **Specified by:**: `getRevision` in interface `RevisionHandler`
    :   **Returns:**: the revision


---


|  |  |  |
| --- | --- | --- |
| **Overview** \| **Package** \| **Class** \| **Tree** \| **Deprecated** \| **Index** \| **Help** \| **Weka's home** | |  |
| **PREV CLASS**   **NEXT CLASS** | **FRAMES**    **NO FRAMES**     **All Classes** |  |
| SUMMARY: NESTED \| FIELD \| CONSTR \| METHOD | DETAIL: FIELD \| CONSTR \| METHOD |  |


---
